# Identification and validation of feature genes using a ferroptosis-informed analytical framework in medulloblastoma

**DOI:** 10.3389/fped.2026.1891716

**Published:** 2026-09-28

**Authors:** Xin Chen, Sen Li, Kunfang Yang, Yu Liu, Qijia Zhan

**Affiliations:** 1 Department of Ward No.6A, Children's Hospital of Shanghai, Shanghai, China; 2 Department of Neurosurgery, Children's Hospital of Shanghai, Shanghai, China; 3 Department of Pediatrics, Shanghai United Family Hospital, Shanghai, China

**Keywords:** candidate biomarker genes, diagnostic biomarkers, ferroptosis, machine learning, medulloblastoma, WGCNA

## Abstract

**Background:**

This study aimed to identify potential biomarker signatures for medulloblastoma using a ferroptosis-informed analytical framework.

**Methods:**

Transcriptomic data from GEO datasets were used to identify differentially expressed ferroptosis-associated genes (DEFeGs) in medulloblastoma by intersecting differentially expressed genes with the FerrDb V2 database. A ferroptosis-related gene (FeRG) score was calculated using ssGSEA. Hub genes correlated with the FeRG score were identified using WGCNA. Candidate feature genes were selected using machine learning algorithms. A nomogram was constructed to predict the probability of medulloblastoma diagnosis. Immune infiltration analysis, drug sensitivity prediction, and qRT-PCR validation of feature gene expression in human samples were performed.

**Results:**

A total of 36 DEFeGs associated with medulloblastoma were identified. WGCNA identified 114 hub genes, of which 58 were differentially expressed. Machine learning algorithms identified *CEND1, LRP1B,* and *FEZ1* as candidate feature genes. A nomogram based on these genes showed high diagnostic performance, which was supported by ROC analysis (all AUCs > 0.85). Nineteen immune cell types differed significantly between the medulloblastoma and control groups. Additionally, *FEZ1, CEND1,* and *LRP1B* showed positive correlations with sensitivity to SGX-523, sabutoclax, and AZD-1208, respectively, whereas all three genes were negatively correlated with sensitivity to allopurinol. The three feature genes were consistently downregulated in medulloblastoma across public datasets and the validation cohort.

**Conclusion:**

This study identified *FEZ1, CEND1,* and *LRP1B* as potential candidate biomarker genes for medulloblastoma through a ferroptosis-informed analytical framework.

## 1 Introduction

Medulloblastoma is the most common malignant pediatric brain tumor arising in the cerebellum and remains one of the leading causes of cancer-related mortality in children (1). Based on molecular characteristics, medulloblastoma is classified into four major subgroups: WNT, which is associated with the most favorable prognosis; SHH, characterized by Hedgehog pathway activation and intermediate risk; and Groups 3 and 4, which generally exhibit a higher metastatic potential and poorer outcomes (2). Current treatment consists of maximal safe surgical resection followed by craniospinal irradiation and multi-agent chemotherapy, resulting in 5-year survival rates ranging from 50% to 90%, depending on molecular subgroup and risk stratification (3). Despite these advances, survivors frequently experience long-term neurocognitive, neurological, and endocrine sequelae resulting from intensive multimodal therapy (4, 5). Therefore, the identification of reliable biomarker candidates and novel therapeutic targets remains a clinical priority to facilitate earlier diagnosis and improve treatment strategies while minimizing long-term toxicities.

Ferroptosis is a regulated form of cell death characterized by iron-dependent lipid peroxidation and oxidative membrane damage. Increasing evidence indicates that dysregulation of ferroptosis contributes to tumor progression, therapeutic resistance, and alterations in the tumor microenvironment (6, 7). Pharmacological induction of ferroptosis has been reported to enhance the antitumor efficacy of conventional therapies in several malignant brain tumors, such as glioblastoma, neuroblastoma, meningioma, and glioma (8, 9). In medulloblastoma, activation of ferroptosis has been shown to sensitize SHH tumor cells to radiotherapy (10). Moreover, ferroptosis-associated transcriptional signatures have demonstrated diagnostic and prognostic value in gliomas (11–13). However, systematic investigations of ferroptosis-associated molecular signatures in medulloblastoma remain limited.

In the present study, a ferroptosis-informed analytical framework integrating transcriptomic profiling, weighted gene co-expression network analysis (WGCNA), and machine learning was employed to identify candidate biomarker genes for medulloblastoma. The resulting feature genes were associated with ferroptosis-related transcriptional patterns and may provide new insights into the molecular landscape of medulloblastoma while serving as candidates for future mechanistic investigations.

## 2 Materials and methods

### 2.1 Study design

This study integrated transcriptomic analysis and machine learning within a ferroptosis-informed analytical framework to identify candidate biomarker genes for medulloblastoma. Gene expression profiles from four Gene Expression Omnibus (GEO) datasets were merged and batch-corrected to identify differentially expressed genes (DEGs) between tumor and control samples. Ferroptosis-associated DEGs (DEFeGs) were obtained by intersecting DEGs with ferroptosis-related genes from the FerrDb V2 database.

A ferroptosis-related gene (FeRG) score was calculated using single-sample gene set enrichment analysis (ssGSEA). WGCNA was performed to identify gene modules associated with the FeRG score, followed by hub gene selection. Candidate feature genes were selected using Least Absolute Shrinkage and Selection Operator (LASSO), Support Vector Machine-Recursive Feature Elimination (SVM-RFE), and Random Forest algorithms. Immune infiltration and drug-gene correlations were assessed, and the expression of feature genes was validated in human medulloblastoma tissue samples using qRT-PCR. The study workflow is summarized in Figure 1.

**Figure 1:**
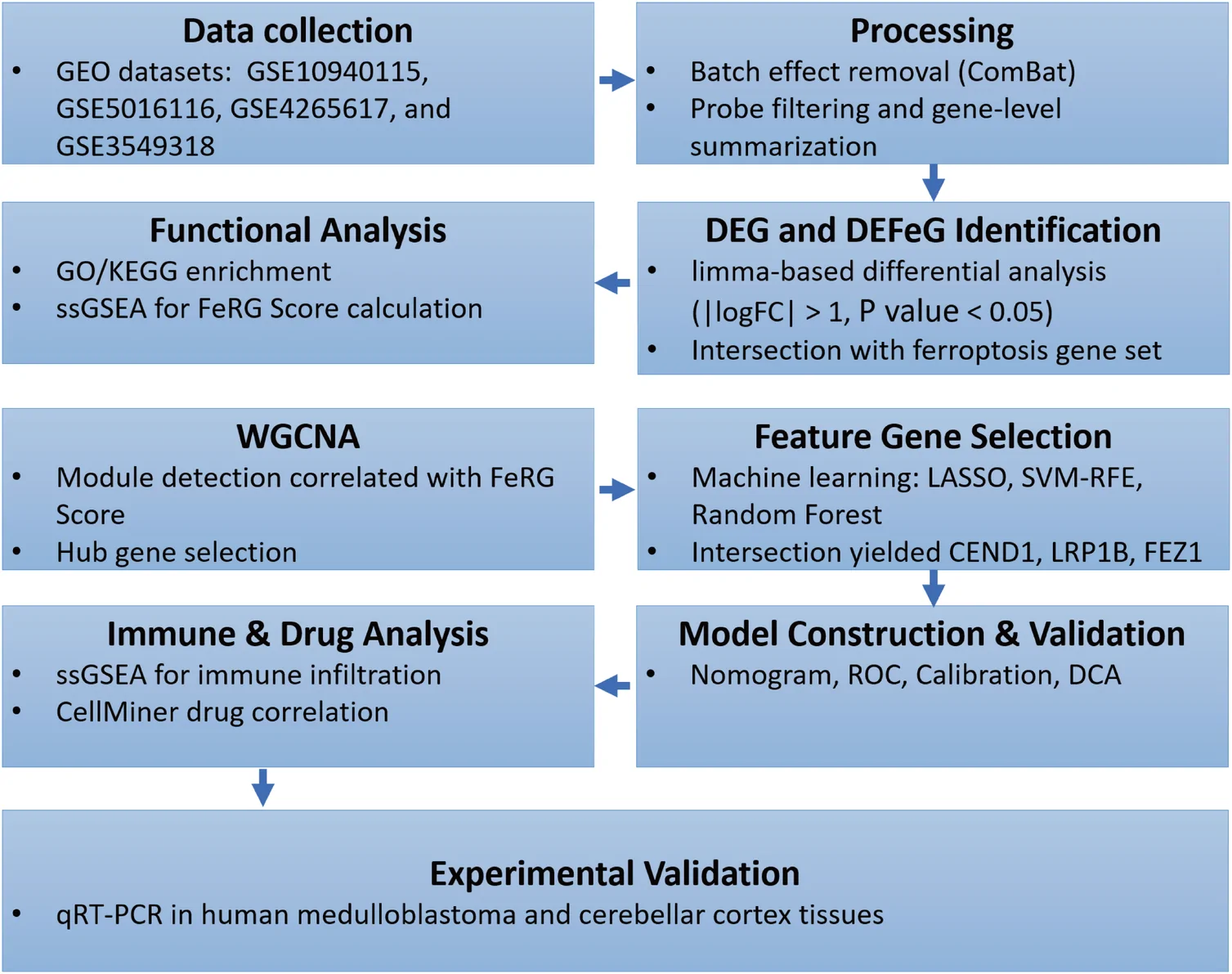
Study workflow. GEO datasets were merged and batch-corrected using ComBat. DEGs were identified using |logFC| > 1 and *P* value < 0.05 as the initial screening threshold, and DEFeGs were obtained by intersecting DEGs with ferroptosis-related genes from FerrDb V2. A FeRG score was calculated by ssGSEA, and WGCNA was performed to identify modules correlated with the FeRG score. Hub genes were selected based on |MM| > 0.6 and |GS| > 0.5 Candidate feature genes were identified by LASSO, SVM-RFE, and Random Forest algorithms, and a nomogram was constructed. Immune infiltration, drug sensitivity, and qRT-PCR validation were performed.

### 2.2 Data retrieval and processing

Transcriptomic expression profiles were obtained from the GEO datasets GSE109401 (14), GSE50161 (15), GSE42656 (16), and GSE35493 (17) (https://www.ncbi.nlm.nih.gov/geo/). GSE109401 exclusively contained medulloblastoma and normal cerebellum samples. Although GSE50161, GSE42656, and GSE35493 included multiple brain tumor types, only medulloblastoma and normal control samples were used in this study. Sample characteristics are summarized in Table 1. These four datasets were merged for candidate biomarker identification, and batch effects were corrected using the ComBat function from the sva package (18). For each dataset, probe IDs were mapped to gene symbols, and probes without gene annotations were removed. The median expression value was used when multiple probes mapped to the same gene. A reference set of ferroptosis-associated genes was obtained from the FerrDb V2 database (http://www.zhounan.org/ferrdb/current/) and was used to define DEFeGs by intersecting with the identified DEGs.

**Table 1:** Summary of gene expression datasets used for differential expression analysis.

Dataset	Platform	Sample types	Sample sizes	Cancer type	Subtypes (n)	Age range (years)	Sex distribution
GSE109401	GPL16686	Cerebellum tissues	19 tumor, 5 normal	Medulloblastoma	WNT (5), SHH (5), Group 3 (5), Group 4 (4)	1–15	11 M, 6 F, 2 unknown
GSE50161^a^	GPL570	Brain tissues	22 tumor, 13 normal	Medulloblastoma	Not reported	3–15	Not reported
GSE42656^a,b^	GPL6947	Cerebellum tissues	9 tumor, 8 normal	Medulloblastoma	Not reported	Pediatric	Not reported
GSE35493^a,b,c^	GPL570	Brain tissues	21 tumor, 9 normal	Medulloblastoma	Not reported	Pediatric	Not reported

a Only medulloblastoma samples were included from GSE50161.

a,b Only medulloblastoma tumor samples were included from GSE42656; normal controls were derived from adult cerebellum tissue.

a,b,c Only medulloblastoma samples were included from GSE35493.

### 2.3 Human sample collection

Tissue specimens for qRT-PCR validation were collected from five children with primary cerebellar tumors (four boys and one girl; age range, 4–12 years) who underwent surgical resection at the Children's Hospital of Shanghai between May 2021 and August 2023. Postoperative histopathological examination confirmed medulloblastoma (WHO grade IV) in all cases. The specimens comprised five medulloblastoma tumor tissues and three normal cerebellar cortex tissues obtained during surgery. Written informed consent was obtained from the legal guardians of all participants prior to tissue collection. The study was approved by the Ethics Review Committee of Children's Hospital of Shanghai/Shanghai Children's Hospital, Shanghai Jiao Tong University (Approval No. 2023R084-E01).

### 2.4 Identification of DEFeGs

The GSE109401, GSE50161, GSE42656, and GSE35493 datasets were merged, and DEGs between medulloblastoma and control samples were identified using the limma package in R (19). Genes with |logFC| > 1 and *P* value < 0.05 were considered DEGs for initial screening to avoid prematurely excluding potentially relevant genes. DEGs overlapping with the ferroptosis-associated gene set obtained from the FerrDb V2 database were defined as DEFeGs and were used for downstream ferroptosis-related analyses. The final candidate feature genes identified through subsequent analyses were further examined for differential expression significance, and all three feature genes satisfied the more stringent criterion of FDR-adjusted *P* < 0.05.

### 2.5 Functional enrichment analysis and ssGSEA

Gene Ontology (GO; http://www.geneontology.org/) and Kyoto Encyclopedia of Genes and Genomes (KEGG; https://www.kegg.jp/) enrichment analyses were performed using the R clusterProfiler package in R to investigate the biological functions and pathways associated with the DEFeGs (20). Adjusted *P* values were calculated using the Benjamini-Hochberg method, and terms with adjusted *P* < 0.05 were considered significantly enriched. A ferroptosis-related gene (FeRG) score was calculated for each sample using ssGSEA implemented in the GSVA package (21).

### 2.6 WGCNA

WGCNA was performed using the WGCNA R package (22) to identify gene modules associated with the FeRG score. Only medulloblastoma samples from the merged datasets were included in the network analysis. Genes with the top 50% variance across samples were selected for network construction. After removing outlier samples, the optimal soft-thresholding power (*β*) was determined using the PickSoftThreshold function based on the scale-free topology criterion. A soft-thresholding power of 6 was selected to achieve a scale-free topology fit index (R^2^) > 0.8, which balances network connectivity and sparsity for robust module detection (23). The networkType, deepSplit, and pamStage parameters were set to “signed”, 2, and TRUE, respectively (24). A weighted adjacency matrix was constructed and transformed into a topological overlap matrix. Gene modules containing at least 30 genes were identified by hierarchical clustering, and modules with a dissimilarity < 0.25 were merged. Module eigengenes were correlated with the FeRG score to identify ferroptosis-associated modules. Hub genes were defined based on |MM| > 0.6 and |GS| > 0.5 Hub genes that were also differentially expressed were retained for subsequent machine learning analyses.

### 2.7 Identification of feature genes

Differentially expressed hub genes were used to identify candidate feature genes in the merged dataset. SVM-RFE, a backward feature selection algorithm based on support vector machines with a radial basis function kernel, was implemented using the caret package in R (25). LASSO was performed using the glmnet package (26). A logistic regression model was fitted, and the optimal feature set was selected using the one-standard-error (1-SE) criterion, with genes exhibiting non-zero regression coefficients retained as candidate features. Random Forest analysis was performed to rank gene importance, and genes with a Gini importance score > 1 were considered informative features. The intersection of the feature genes identified by the three algorithms was used to define the final set of candidate feature genes.

### 2.8 Nomogram construction and evaluation

A nomogram was constructed based on the expression levels of the selected candidate feature genes using the rms package in R (27). The merged and batch-corrected gene expression profiles from the four GEO datasets were used as input. For each sample, a risk score was calculated by weighting the normalized expression values of the feature genes according to their corresponding logistic regression coefficients. The total score was then converted into the estimated probability of medulloblastoma using the logistic regression model. The predictive performance of the nomogram was evaluated using calibration curves generated with the calibrate function in the rms package (28) and decision curve analysis (DCA) performed using the rmda package (29).

### 2.9 Immune infiltration analysis

The relative enrichment of 28 immune cell subtypes was estimated using ssGSEA as previously described (30). ssGSEA was implemented using the GSVA package in R (21) to calculate enrichment scores for each immune cell subtype in individual samples. Differences in immune cell abundance between the medulloblastoma and control groups were evaluated using the Wilcoxon rank-sum test. Pearson correlation analysis was performed to assess the associations between candidate feature gene expression and immune cell enrichment scores. Given the exploratory nature of this analysis, nominal *P* values (*P* < 0.05) were used as a screening threshold without formal multiple-testing correction.

### 2.10 Drug sensitivity prediction

Associations between candidate feature gene expression and drug sensitivity were evaluated using gene expression data from the NCI-60 cell line panel and corresponding drug activity data available in the CellMiner database (https://discover.nci.nih.gov/cellminer/home.do). Pearson correlation analysis was performed to assess the relationship between gene expression and drug sensitivity. As this analysis was exploratory, correlations with nominal *P* < 0.05 were considered without adjustment for multiple comparisons. Gene-drug associations were visualized using Cytoscape.

### 2.11 qRT-PCR

Five frozen medulloblastoma tissue specimens and three cerebellar cortex tissue specimens were included for qRT-PCR validation. Total RNA was extracted using the RNeasy Mini Kit (Qiagen, Valencia, CA). One microgram of total RNA was reverse transcribed into cDNA using a reverse transcription kit (Accurate Biology, Hunan, China). Gene expression was quantified by qRT-PCR using the Bio-Rad CFX96 Real-Time PCR System (Bio-Rad, Hercules, CA, USA). Gene-specific primers were synthesized by Sangon Biotech (Shanghai, China). *GAPDH* was used as the internal reference gene. Primer sequences were as follows: *FEZ1*: forward 5'-GCTCTGAATGGCAACTGCTCTG-3' and reverse 5'-CTCCTCAATTACCTGATCTGCTGTG-3' (129 bp); *LRP1B*: forward 5'-GTTCAGATGTTCCAGTGGCAGATG-3' and reverse 5'-CCGTTACAACCAGCAGGAGAATG-3' (135 bp); *CEND1*: forward 5'-CCTTCTCAACAACCACAGCAACC-3' and reverse 5'-ACTGGCAGCCTCATCTTCCTC-3' (115 bp). Relative gene expression was calculated using the 2^−*ΔΔ*Ct^ method.

### 2.12 Statistical analysis

Statistical analysis was performed using R software (version 4.3.0). Principal component analysis (PCA) was conducted using the FactoMineR and factoextra packages. Heatmaps, Venn diagrams, and receiver operating characteristic (ROC) curves were generated using the pheatmap, ggvenn, and pROC packages, respectively. Other graphical visualizations were produced using ggplot2 or the base plot package. Pearson correlation analysis was performed to evaluate associations between continuous variables. For comparisons between two independent groups, the two-sided Mann–Whitney U test (Wilcoxon rank-sum test) was used. For qRT-PCR validation, technical replicates were summarized within each biological specimen, and independent biological specimens were used as the statistical units. A two-sided *P* value < 0.05 was considered statistically significant.

## 3 Results

### 3.1 Identification of DEGs in medulloblastoma

Gene expression profiles from four datasets (GSE109401, GSE50161, GSE42656, and GSE35493) were integrated to identify DEGs between medulloblastoma and control samples. The merged datasets included 67 medulloblastoma samples and 35 control samples. Before batch effect correction, PCA revealed distinct clustering according to dataset origin (Figures 2A,C). Following ComBat correction, samples exhibited improved clustering consistency across datasets (Figures 2B,D). Using the thresholds of |logFC| > 1 and *P value* < 0.05 for initial screening, a total of 1,484 DEGs were identified, including 616 upregulated and 868 downregulated genes (Figure 2E; Supplementary Table S1). Notably, all three final feature genes also satisfied the more stringent criterion of FDR-adjusted *P* < 0.05.

**Figure 2:**
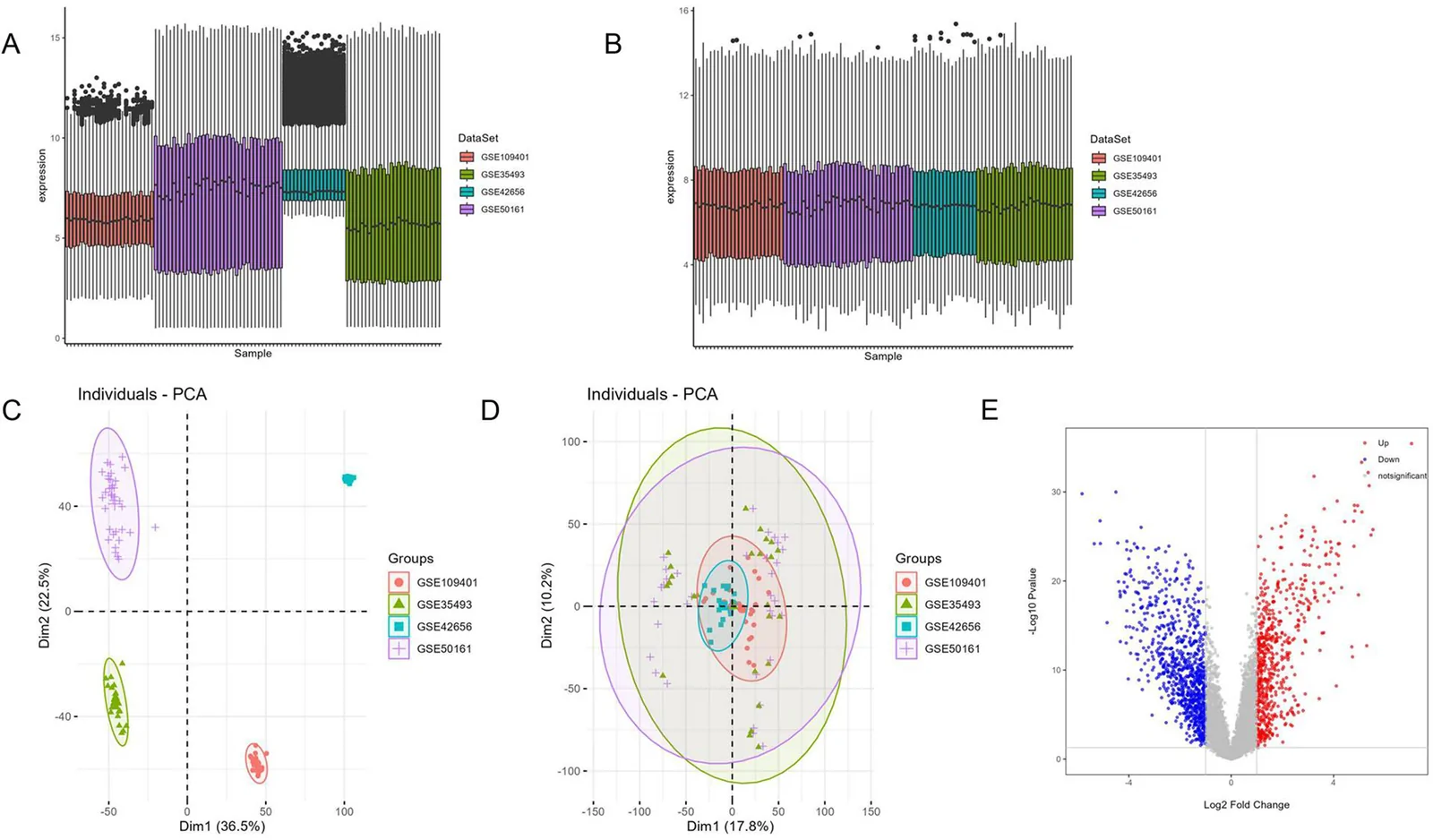
Identification of differentially expressed genes between medulloblastoma and control groups. **(A,C)** Box plots and Principal Component Analysis (PCA) before batch effect removal show different expression patterns from different datasets. **(B,D)** Box plots and PCA after batch effect removal demonstrate consistent expression patterns among different datasets. **(E)** Volcano plot illustrates differentially expressed genes, where red represents upregulated genes, blue represents downregulated genes, and gray represents non-significant changes.

### 3.2 Identification and functional enrichment analysis of DEFeGs

DEFeGs were identified by intersecting the DEGs with the ferroptosis-associated gene set obtained from the FerrDb V2 database, resulting in 11 upregulated and 25 downregulated DEFeGs (Figures 3A,B). Based on the expression profiles of these 36 DEFeGs, a FeRG score was calculated using ssGSEA. The FeRG score was significantly lower in medulloblastoma than in control samples (Figure 3C). A heatmap demonstrated that most DEFeGs were downregulated across the merged datasets (Figure 3D).

**Figure 3:**
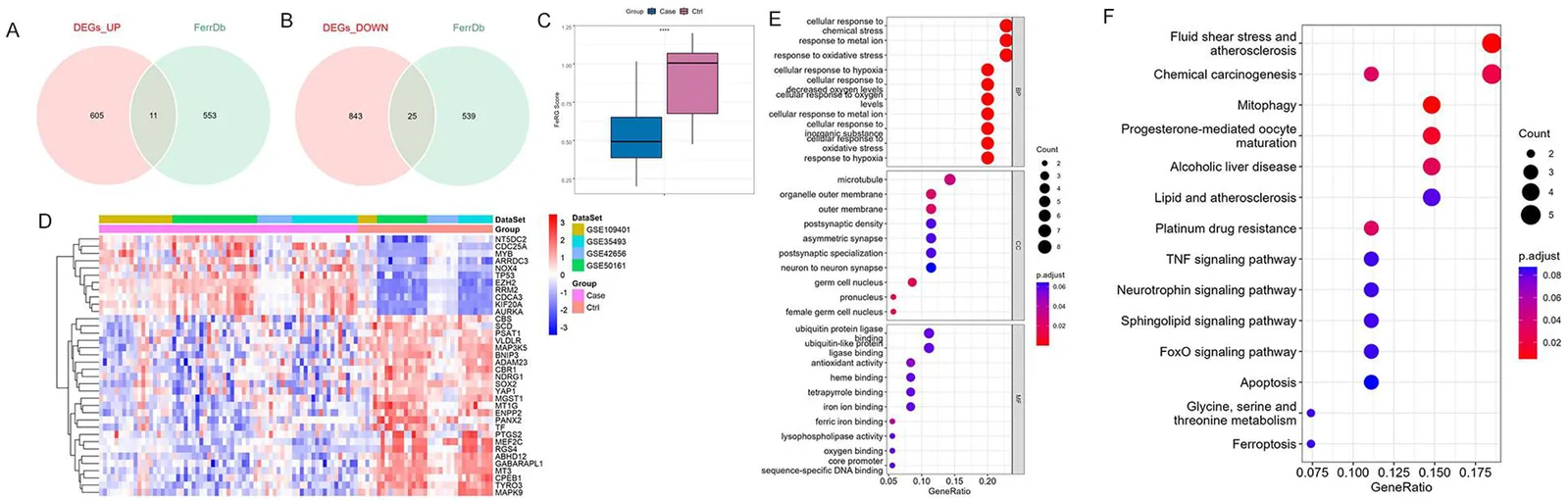
Identification and characterization of differentially expressed ferroptosis-related genes (DEFeGs). **(A,B)** Venn diagrams depict the intersection of upregulated and downregulated DEGs with ferroptosis-related genes. **(C)** Box plots illustrate the difference in FeRG Scores between the medulloblastoma group and the control group. **(D)** Heatmap displays the expression patterns of DEFeGs across various datasets. **(E)** GO analysis. **(F)** KEGG analysis. BP, biological processes; CC, cellular components; MF, molecular functions.

GO enrichment analysis indicated that the DEFeGs were significantly enriched in biological processes related to cellular responses to hypoxia and metal ion homeostasis, cellular components such as the organelle outer membrane and pronucleus, and molecular functions including ferric iron binding (Figure 3E; Supplementary Table S2). These enriched terms are broadly associated with oxidative stress and iron homeostasis, biological processes closely related to ferroptosis. KEGG pathways analysis revealed enrichment in mitophagy, platinum drug resistance, and chemical carcinogenesis (Figure 3F; Supplementary Table S3). Although these pathways are not canonical ferroptosis pathways, they are involved in biological processes that have been reported to interact with ferroptosis, including mitochondrial homeostasis, oxidative stress responses, and chemoresistance (31). These findings suggest that ferroptosis-associated transcriptional alterations in medulloblastoma are linked to broader changes in cellular stress responses and metabolic regulation.

### 3.3 Identification of core modules and hub genes

WGCNA was performed on the medulloblastoma samples from the merged datasets to identify gene modules associated with the FeRG score. After sample clustering, two outlier samples were excluded (Figure 4A). A soft-thresholding power (*β*) of 6 was selected to achieve a scale-free topology fit index (R^2^) of approximately 0.8 (Figure 4B). The clustering dendrogram and corresponding distribution of FeRG scores are shown in Figure 4C. Hierarchical clustering identified 23 co-expression modules (Figure 4D). Among these, the black module exhibited the strongest positive correlation with the FeRG Score (R = 0.85; Figure 4E). Using the thresholds of |GS| > 0.5 and |MM| > 0.6, 114 hub genes were identified within the black module (Figure 4F). Of these, 58 were differentially expressed between the medulloblastoma and control samples and were retained for subsequent machine learning analysis (Supplementary Table S4).

**Figure 4:**
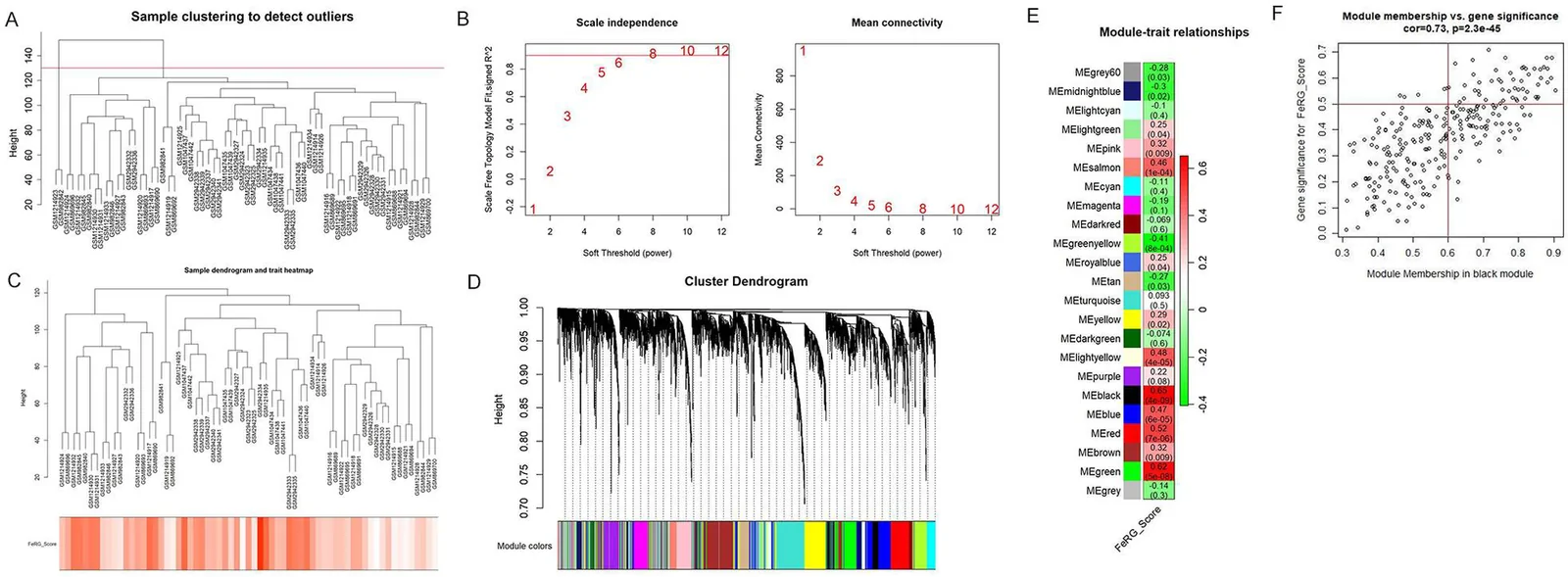
Identification of hub DEFeGs. **(A)** Sample clustering with removal of outliers. **(B)** Soft threshold *β* selection. **(C)** Sample clustering along with the distribution of FeRG Scores. **(D)** Co-expression module clustering. Different colors represent various co-expression modules. **(E)** Correlation between modules and FeRG Score. **(F)** Hub gene selection within the black module.

### 3.4 Identification of feature genes

Three machine learning algorithms were applied to the 58 differentially expressed hub genes identified from the WGCNA black module to select candidate feature genes. In the Random Forest model, the lowest classification error was achieved with a mtry value of 3 (Figure 5A), and the error rate stabilized when the number of trees (ntree) exceeded 100 (Figure 5B). Based on a Gini importance score > 1, 13 genes were retained as candidate features (Figure 5C). LASSO regression identified 14 genes with non-zero coefficients at the optimal lambda value (Figures 5D,E). The SVM-RFE model achieved the highest classification accuracy using 6 genes (Figure 5F). The intersection of the three algorithms identified *CEND1, LRP1B,* and *FEZ1* as the final candidate feature genes (Figure 5G). Evaluation of the four individual GEO datasets demonstrated consistent downregulation of all three genes in medulloblastoma compared with control samples (Supplementary Figure S1).

**Figure 5:**
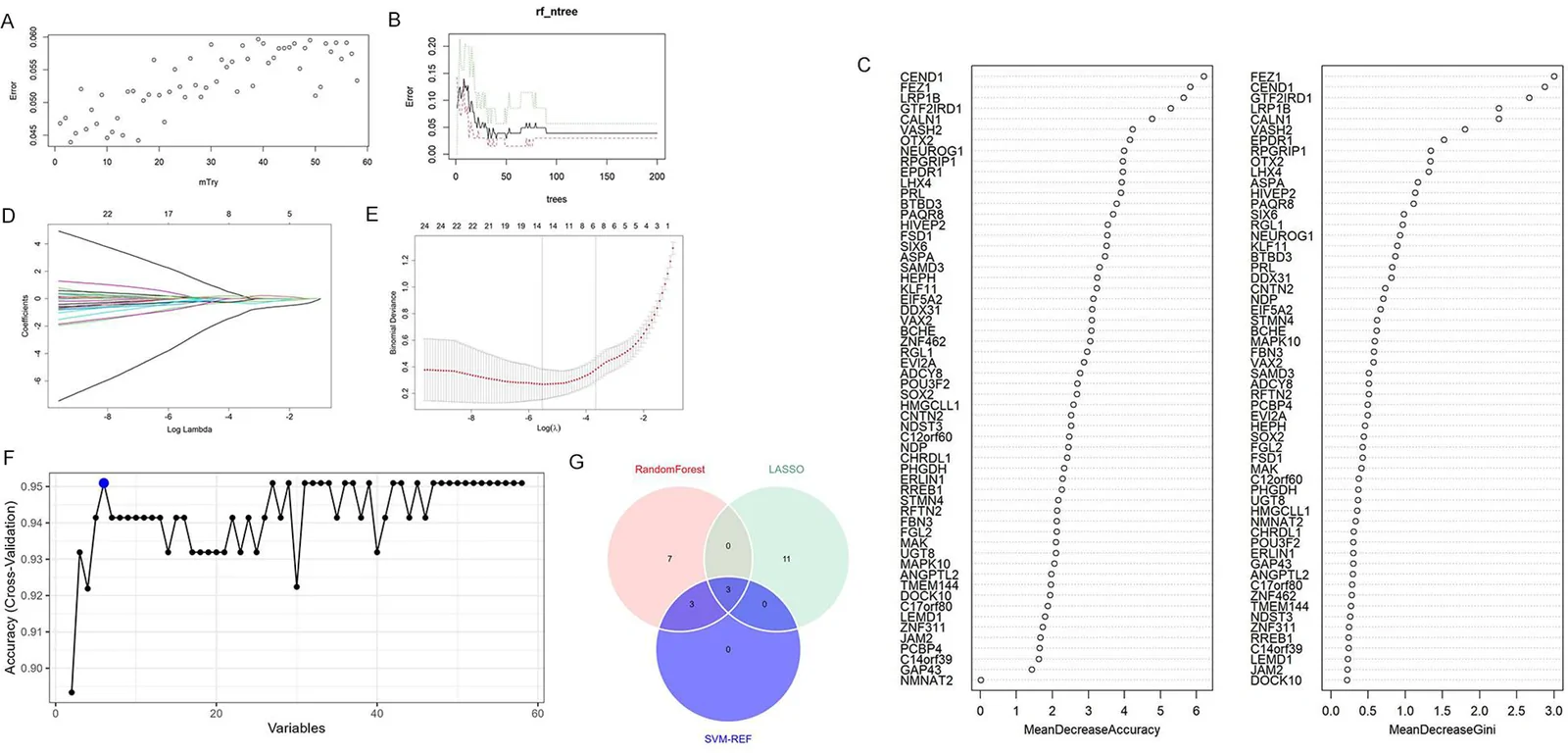
Identification of featured genes. **(A,B,C)** Relationship between the number of features, the number of classification trees, and error rate in the random forest model, along with feature importance ranking. **(D,E)** Feature selection using the LASSO regression model. **(F)** Relationship between SVM-RFE features and model accuracy. **(G)** Venn diagram showing the intersection of features identified by the three algorithms.

### 3.5 Discriminative performance of feature genes

A nomogram incorporating the three candidate feature genes was constructed to estimate the probability of medulloblastoma (Figure 6A). For each sample, the total score was calculated by summing the points assigned according to the expression levels of each feature gene. DCA demonstrated that the combined model provided greater net clinical benefit than any individual feature gene (Figure 6B). Calibration analysis showed good agreement between the predicted and observed probabilities of medulloblastoma (Figure 6C). ROC analysis demonstrated consistent discriminative performance of the three-gene signature across both the merged dataset and the four individual datasets (all AUC > 0.85; Figures 6D–H), indicating that the discriminative ability of the signature is reproducible across independently generated cohorts. Furthermore, the consistent downregulation of all three feature genes in each individual dataset (Supplementary Figure S1) and the significant expression changes confirmed by qRT-PCR in an independent human tissue cohort (Figure 7) provide complementary lines of evidence supporting the robustness of the identified signature. These findings support the potential of the three-gene signature in distinguishing medulloblastoma from normal cerebellar tissue, although fully independent external validation in larger cohorts remains warranted.

**Figure 6:**
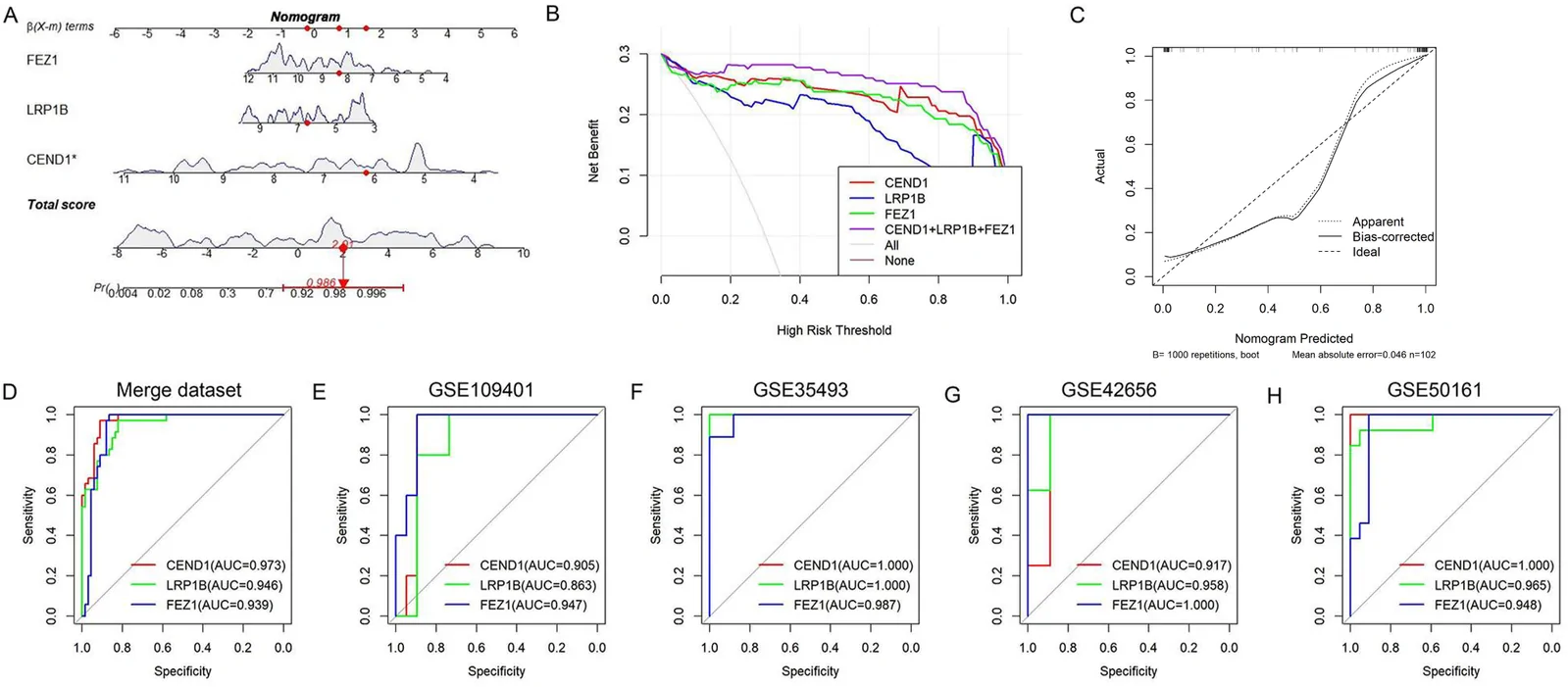
Construction and evaluation of the medulloblastoma diagnostic nomogram. **(A)** Nomogram for predicting the probability of medulloblastoma vs. normal cerebellar tissue based on the expression levels of the three feature genes. For each gene, points are assigned according to its expression value; the total score is the sum of individual points, and the corresponding probability of medulloblastoma is obtained by drawing a vertical line from the total score to the probabilit*y* axis (the example shown yields an estimated probability of 98.6%). **(B)** Decision curve analysis (DCA) evaluating the net clinical benefit of the three-gene signature across a range of threshold probabilities. **(C)** Calibration curve showing agreement between the nomogram-predicted probability and the observed probability of medulloblastoma. **(D–H)** ROC curves for the three-gene signature in the merged dataset and in each individual GEO dataset (GSE109401, GSE50161, GSE42656, and GSE35493), with AUC values annotated.

**Figure 7:**
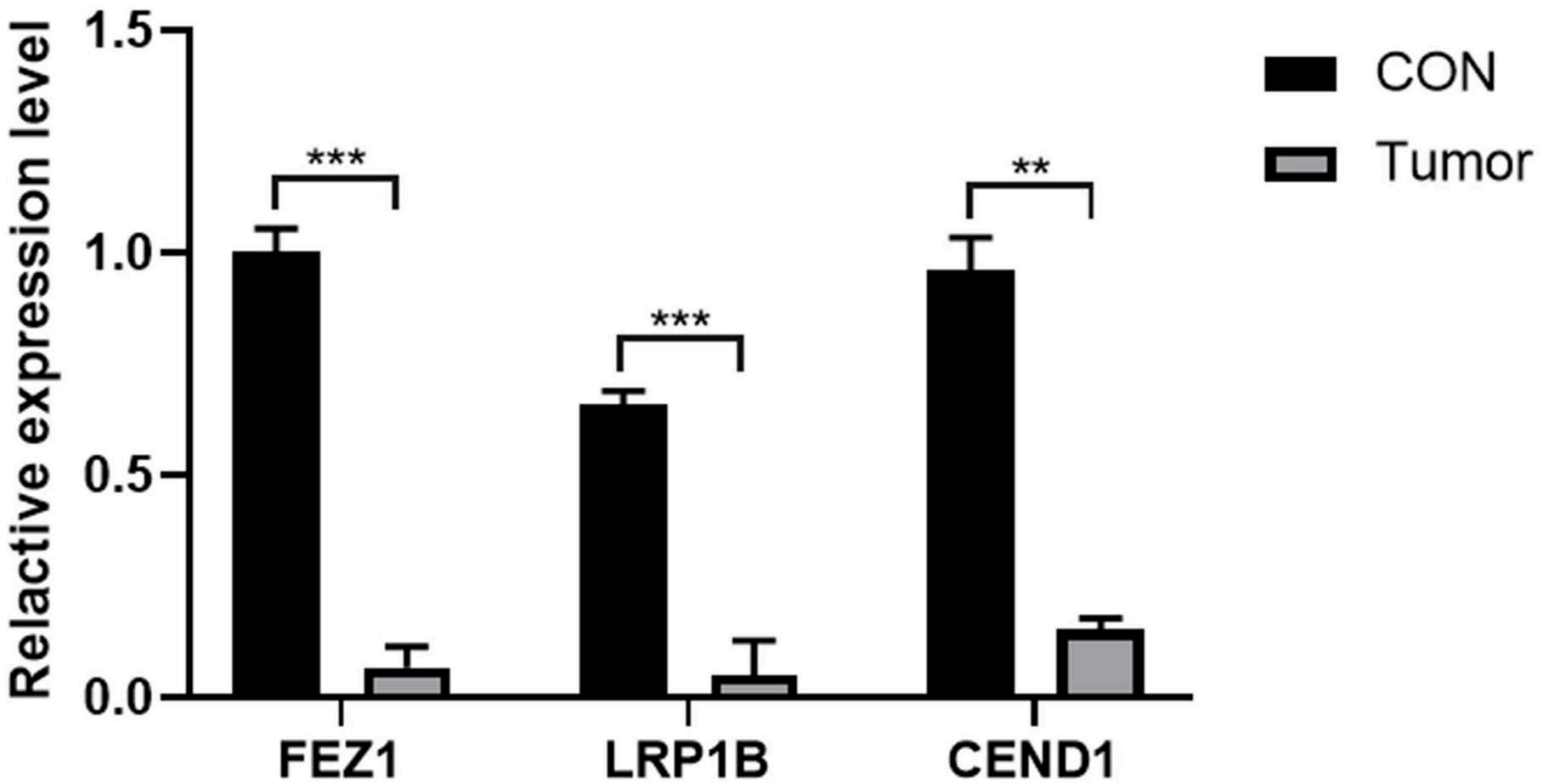
Expression levels of feature genes in medulloblastoma tissue and normal cerebellar cortex tissue. qRT-PCR was performed on tumor tissue (*n* = 5) and normal cerebellar cortex tissue samples (*n* = 3) to determine mRNA levels of feature genes. Data are expressed as the mean ± standard deviation. Statistical comparisons were performed using the two-sided Mann–Whitney U test based on independent biological samples. ** *P* < 0.01, ***P < 0.001.

### 3.6 Immune infiltration analysis

Given the growing evidence linking ferroptosis to immune regulation in medulloblastoma (32), immune cell infiltration was evaluated using ssGSEA. Among the 28 immune cell types analyzed, 19 differed significantly between the medulloblastoma and control groups, including activated CD4 T cells, memory B cells, plasmacytoid dendritic cells, eosinophils, immature dendritic cells, gamma delta T cells, monocytes, T follicular helper (Tfh) cells, CD56^bright^ natural killer cells, effector memory CD4T cells, type 2 T helper cells, CD56^dim^ natural killer cells, activated dendritic cells, central memory CD4 T cells, neutrophils, macrophages, myeloid-derived suppressor cells (MDSCs), type 1 T helper cells, and type 17 T helper cells (Supplementary Figure S2A). The distribution of the 28 immune cell subtypes across samples is shown in Supplementary Figure S2B. Correlation analysis showed that *CEND1, FEZ1,* and *LRP1B* exhibited similar patterns of association with multiple immune cell subtypes (Supplementary Figures S2C–E). These findings suggest that the three candidate feature genes are associated with the immune microenvironment in medulloblastoma and warrant further investigation into their potential immunological relevance.

### 3.7 Feature gene-drug correlation analysis

To explore the potential pharmacological relevance of the candidate feature genes, associations between gene expression and drug sensitivity were evaluated using the NCI-60 cancer cell line panel. Pearson correlation analysis showed that *FEZ1, CEND1,* and *LRP1B* exhibited the strongest positive correlations with sensitivity to SGX-523, sabutoclax, and AZD-1208, respectively (*P* < 0.05; Supplementary Figure S3). Conversely, all three genes showed the strongest negative correlations with allopurinol (Supplementary Table S5). These findings suggest potential associations between the feature genes and drug sensitivity; however, further experimental validation is required to determine their therapeutic relevance in medulloblastoma.

### 3.8 Verification of feature gene alterations in human samples

The expression levels of the three candidate feature genes were validated by qRT-PCR in medulloblastoma and normal cerebellar cortex tissue samples. Compared with normal cerebellar cortex tissue, *FEZ1, LRP1B,* and *CEND1* were significantly downregulated in medulloblastoma tissue (Figure 7), consistent with the bioinformatic findings.

## 4 Discussion

This study identified 1,484 DEGs in medulloblastoma across four datasets. Among these, 36 were DEFeGs, most of which were downregulated in medulloblastoma. WGCNA identified a black module that was significantly correlated with the FeRG score, of which 58 of the 114 hub genes were differentially expressed. Machine learning analyses further identified three candidate feature genes, *CEND1, LRP1B,* and *FEZ1*. These genes also exhibited similar association patterns with multiple immune cell types and were associated with sensitivity to specific small-molecule compounds in the NCI-60 cell line panel, suggesting their potential relevance to the immune microenvironment and pharmacological profiling of medulloblastoma.

Previous studies have shown that *CEND1* regulates neuronal differentiation (33) and has been associated with glioma diagnosis (34) and temozolomide resistance (35). *LRP1B* functions as a putative tumor suppressor and has been implicated in medulloblastoma and glioblastoma progression (36–39). *FEZ1* has also been reported to be downregulated in medulloblastoma (40). In the present study, *CEND1, LRP1B,* and *FEZ1* were identified as candidate feature genes by multiple machine learning algorithms. Their expression patterns were consistently observed across multiple datasets and confirmed by qRT-PCR in human tissues. Although these genes were identified through a ferroptosis-informed analytical framework, they are not established regulators of ferroptosis. Therefore, our findings indicate a potential association with ferroptosis-related transcriptional alterations but do not establish a causal role for these genes in ferroptosis.

The immune infiltration analysis identified significant differences in 19 immune cell populations between medulloblastoma and control tissues. In addition, *CEND1, LRP1B,* and *FEZ1* exhibited similar patterns of association across multiple immune cell types, suggesting that these genes share similar associations with the immune microenvironment in medulloblastoma. Previous studies have reported correlations between *LRP1B* mutations and immune infiltration in several cancers (41, 42). *CEND1* has also been associated with immune infiltration in glioma (43). These findings suggest that the identified feature genes may be associated with the immune microenvironment of medulloblastoma; however, the underlying mechanisms require further investigation.

Drug sensitivity analysis using the NCI-60 cell line panel identified significant associations between the three feature genes and specific small-molecule compounds. *FEZ1, CEND1, and LRP1B* were positively associated with sensitivity to SGX-523, sabutoclax, and AZD-1208, respectively, whereas all three genes were negatively associated with allopurinol. SGX-523 targets c-Met, which has been implicated in medulloblastoma (44, 45). Sabutoclax and AZD-1208 inhibit Bcl-2 and PIM kinase, respectively (46, 47). Although these findings are based on correlations derived from cancer cell lines and require experimental validation, they may provide a basis for future therapeutic investigation.

While the study offers valuable insights, several limitations should be noted. The study relied on publicly available gene expression datasets, which may not fully capture the biological heterogeneity of medulloblastoma. Although batch effects were corrected using the ComBat algorithm, residual technical variation introduced by integrating datasets from different microarray platforms cannot be completely excluded. qRT-PCR validation was performed in a relatively small cohort due to the limited availability of pediatric medulloblastoma specimens, and validation in larger independent cohorts is needed to support these findings. In addition, prognostic validation could not be performed because survival information was unavailable for the integrated datasets, and future studies using cohorts with comprehensive clinical follow-up are warranted. Correlations between gene expression, immune infiltration, and drug sensitivity are associative and require biological validation. The immune infiltration and drug-sensitivity analyses were exploratory and used nominal *P* values without correction for multiple comparisons; therefore, these findings should be interpreted as hypothesis-generating. Protein-level validation was not performed, and the biological functions of *CEND1, LRP1B,* and *FEZ1*, including their relationship with ferroptosis, require further investigation. The translational potential of the proposed diagnostic and therapeutic implications also requires clinical validation. Additionally, drug sensitivity analyses based on cell lines may not fully reflect the complexity of human tumors *in vivo*. Although medulloblastoma comprises four molecular subgroups, only GSE109401 included subtype annotations. Due to the small and imbalanced sample sizes within each subgroup, this dataset was insufficient for robust subgroup-specific evaluation of the gene signature. Future analyses incorporating larger, well-annotated cohorts are needed to determine the diagnostic specificity of the identified feature genes across molecular subtypes. An additional limitation concerns the validation strategy for the diagnostic model. The four GEO datasets used in this study were merged to maximize sample size, given the limited availability of publicly available medulloblastoma transcriptomic datasets with appropriate normal cerebellar controls. While the integrated analysis allowed us to maximize the available data for feature identification and model construction, it also means that the ROC assessments were performed on the same datasets used for model development and therefore do not represent fully independent external validation. We acknowledge that true external validation in entirely separate cohorts is the gold standard; however, the limited sample size and availability of suitable public datasets made it difficult to perform a reliable dataset-level hold-out validation. Instead, we assessed the consistency and generalizability of our findings through multiple complementary approaches, including ROC analysis within each individual dataset (all AUC values > 0.85), consistent downregulation of the three feature genes across all four individual datasets, and experimental validation in an independent human tissue cohort using qRT-PCR. While these lines of evidence support the robustness of the signature, we recognize that they do not substitute for fully independent external validation. Furthermore, the model was trained to distinguish medulloblastoma from normal cerebellar tissue, and its specificity for medulloblastoma relative to other pediatric brain tumors remains to be determined. Future studies with larger, prospectively collected cohorts from diverse institutions are needed to confirm the diagnostic utility and clinical translatability of the three-gene signature.

## 5 Conclusion

In summary, the study identified three candidate feature genes (*CEND1, LRP1B,* and *FEZ1*) through a ferroptosis-informed analytical framework. These genes demonstrated good discriminative performance and were associated with sensitivity to specific small-molecule compounds in the NCI-60 cell line panel. Although these findings provide potential biomarker candidates for medulloblastoma, further validation in larger cohorts and functional studies is required before clinical application.

## Data Availability

The datasets analyzed in this study are publicly available in the NCBI Gene Expression Omnibus (GEO) repository under accession numbers GSE109401, GSE50161, GSE42656, and GSE35493. Further inquiries can be directed to the corresponding author.

## Supplementary material

Supplementary Table S1 mRNA differentially expressed in combined data

Supplementary Table S2 Enrichment results of differentially expressed iron death genes – GO

Supplementary Table S3 Enrichment results of differentially expressed iron death genes – KEGG

Supplementary Table S4 Intersection of differentially expressed genes and WGCNA black module hub genes

Supplementary Table S5 Drug sensitivity analysis using CellMiner

Supplementary Figure S1 Expression of three feature genes in all four datasets.

Supplementary Figure S2 Relationship between Medulloblastoma and Immunity. (A, B) Box plots and heatmaps illustrate the differential abundance of immune cells between medulloblastoma and control groups. (C-E) Correlation between feature genes and immune cells. Longer lines represent stronger correlation, and darker colors indicate more significant correlations.

Supplementary Figure S3 Correlation of feature gene expression and drug sensitivity. Feature gene-drug pairs with *P* < 0.05 in Pearson correlation analysis are shown.
